# Inducible MicroRNA-223 Down-Regulation Promotes TLR-Triggered IL-6 and IL-1β Production in Macrophages by Targeting STAT3

**DOI:** 10.1371/journal.pone.0042971

**Published:** 2012-08-24

**Authors:** Qingyun Chen, Hui Wang, Yang Liu, Yinjing Song, Lihua Lai, Quan Han, Xuetao Cao, Qingqing Wang

**Affiliations:** 1 Institute of Immunology, Zhejiang University School of Medicine, Hangzhou, People's Republic of China; 2 Medical College of Chinese People's Armed Police Force, Tianjin, People's Republic of China; 3 National Key Laboratory of Medical Immunology & Institute of Immunology, Second Military Medical University, Shanghai, People's Republic of China; National Cancer Institute (INCA), Brazil

## Abstract

MicroRNAs are small non-coding RNA molecules that regulate gene expression by either translational inhibition or mRNA degradation. MicroRNAs play pivotal roles in the regulation of both innate and adaptive immune responses, including TLR-triggered inflammatory response. Here we reported that the expression of microRNA-223 (miR-223) was significantly decreased in murine macrophages during activation by lipopolysaccharide (LPS) or poly (I∶C) stimulation. The inducible miR-223 down-regulation resulted in the activation of signal transducer and activator of transcription 3 (STAT3), which is directly targeted by miR-223, thus promoting the production of pro-inflammatory cytokines IL-6 and IL-1β, but not TNF-α. Interestingly, IL-6 was found to be a main factor in inducing the decrease in miR-223 expression after LPS stimulation, which formed a positive feedback loop to regulate IL-6 and IL-1β. Herein, our findings provide a new explanation characterizing the molecular mechanism responsible for the regulation of IL-6 production after TLR-triggered macrophage activation.

## Introduction

Macrophages constitute an important source of many cytokines in immune responses, hematopoiesis, inflammation and many other homeostatic processes [1]. Upon stimulation by micro-organisms, microbial products or endogenous factors (including cytokines), macrophages synthesize and release a large variety of cytokines (e.g. interleukin-1 (IL-1), IL-6, IL-10, tumor necrosis factor-α (TNF-α), interferon (IFN)). Toll-like receptors (TLRs) play a critical role in innate immunity by recognizing a limited but highly conserved set of molecular structures produced by micro-organisms (pathogen-associated molecular patterns, or PAMPs) [2]. TLRs bind microbial factors at the cell surface or in endosomes and subsequently activate cytoplasmic signal transduction pathways to produce inflammatory cytokines. TLR4 recognizes and binds to LPS to trigger a signaling cascade through the MyD88-dependent and/or MyD88-independent signaling pathway, which eventually leads to activation of MAPK and NF-κB, thus resulting in the production of pro-inflammatory cytokines and factors [3].

The signal transducer and activator of transcription (STAT) pathway has also been shown to play roles in the signaling cascades triggered by LPS, IFN and other cytokines [4]–[6]. Among the STAT superfamily, STAT3 is predominantly activated by gp130-acting cytokines (e.g., IL-6, IL-11, oncostatin-M, leukocyte migration inhibition factor (LIF)). Many innate immune cytokines, including the anti-inflammatory IL-10 and pro-inflammatory type I and II IFNs, also activate STAT3, which may account for the fact that STAT3 can mediate both anti-inflammatory and pro-inflammatory responses [7]. The role of STAT3 in facilitating the pro-inflammatory responses of IL-6 and other cytokines has been recently reported. JAK2 and STAT3 have been shown to play pivotal roles in LPS-induced IL-1β and IL-6 production in macrophages [6]. A newly developed STAT3-specific inhibitor (STATtic) was reported to block LPS-mediated STAT3 tyrosine phosphorylation, IL-1β and IL-6 production, with no effect on TNF-α [8]. Recent studies showed that genetic reduction of STAT3 activity in gp130^F/F^: STAT3^+/−^ mice alleviated hypersensitivity and IL-6 levels produced in response to LPS [7]. It is clear that IL-6/STAT3 signaling exerts complex actions in regulating the innate immune response. Activation of STAT3 may promote the IL-6 production, and IL-6 itself can lead to the phosphorylation of STAT3 [9]. The detailed mechanisms of how STAT3 functions towards TLR activation and how STAT3 expression is modulated during inflammatory responses remains to be elucidated. Knowledge of specific molecular events involved in the regulation of IL-6/STAT3 signaling may provide useful insights in understanding macrophage biology and the mechanisms by which STAT3 promotes the pathogenesis of inflammatory diseases.

MicroRNAs (miRNAs) are short (20–23 nucleotides), endogenous, single-stranded RNA molecules that regulate gene expression. They play important roles in biological processes such as cell proliferation and differentiation, development and apoptosis [10]. Recent studies showed that a range of miRNAs have critical functions in the regulation of inflammatory responses in macrophages and monocytes (e.g. miR-155, miR-146a) [11]–[14]. MiR-223 was in the first cadre of miRNAs discovered to be highly expressed in myeloid cells of the bone marrow, where it is mainly expressed in the myeloid, granulocytic and monocytic compartments [15], [16]. Expression of miR-223 is regulated by a combination of factors. Two transcriptional factors (NFI-A and C/EBPα) compete for binding to the miR-223 promoter. MiR-223 itself targets NFI-A, thereby turning off its repressor once it is expressed, forming a positive autoregulatory circuit [17]. Further work has shown that miR-223 gene resembles a “myeloid gene” and might be driven by the myeloid transcription factors, PU.1 and C/EBPs [18]. In cells of the granulocytic lineage, myeloid-specific miR-223 negatively regulates progenitor proliferation, granulocyte differentiation and activation by targeting Mef2c [19]. More recent work has identified that miR-223 targets IKK-α during human monocyte-macrophage differentiation. The down-regulation of miR-223 probably prevents macrophage hyperactivation yet primes the macrophage for certain responses to pro-inflammatory stimuli [20]. These results extend the roles of miR-223 to monocyte/macrophage differentiation, in addition to the previous description of a role in granulocytic differentiation. However, the detailed functions of miR-223 in TLR-triggered cytokine production in macrophages still remain unclear.

Our initial observation of significant down-regulation of miR-223 in murine macrophages upon TLR ligand stimulation (e.g. LPS, poly (I∶C)) prompted us to study the potential effect of miR-223 on macrophage function. MiR-223 overexpression down-regulates IL-6 and IL-1β, but not TNF-α, expression levels in TLR-activated macrophages. And we demonstrated that miR-223 directly targets STAT3 to regulate the pro-inflammatory cytokines. In turn, IL-6, which is triggered by TLR stimulation, is responsible for the down-regulation of miR-223, thus forming a positive regulatory loop for pro-inflammatory cytokine production. Our results uncovered an essential mechanism of miR-223-mediated regulation of TLR-triggered inflammatory cytokines in macrophages.

## Materials and Methods

### Mice and reagents

BALB/c mice (5–6 wks old) were purchased from SIPPR-BK Experimental Animal Ltd Co. (Shanghai, China). Experiments and animal care were performed according to protocols approved by the Zhejiang University Institutional Animal Care and Use Committee. Lipopolysaccharide (LPS) and polyinosinic-polycytidylic acid (poly (I∶C)) were purchased from Sigma (St. Louis, MO). MiR-223 mimics and control, miR-223 inhibitors and inhibitor control, STAT3 small interfering RNA (siRNA) and control siRNA were from GenePharma (Shanghai, China). Anti-β-action and peroxidase (HRP)-labeled secondary antibodies were purchased from Santa Cruz Biotechnology Inc. (Santa Cruz, CA). Anti-STAT3 and anti-p-STAT3 (Y705) were purchased from Cell Signaling. Anti-mouse IL-6 neutralizing antibody was purchased from eBioscience.

### Cell culture and transfection

RAW 264.7 cell lines were obtained from American Type Culture Collection (Manassas, VA) and cultured in DMEM containing 10% FBS. Mouse primary macrophages were obtained and cultured as previous described [21]. 1×10^4^ cells were seeded into 96-well plates and incubated overnight. JetSI-ENDO transfection reagents (Polyplus-transfection, Illkirch, France) were used for the cotransfection of plasmids and RNAs, according to the manufacturer's instructions. 0.5 ml containing 2×10^5^ cells was seeded into each well of 24-well plates and incubated overnight, and then transfected with RNAs using INTERFERin (Polyplus-transfection), according to the manufacturer's instructions.

### Detection of cytokine production

IL-6, TNF-α and IL-1β in the supernatants were measured with ELISA kits (eBioscience) according to the manufacturer's protocols.

### 3′UTR luciferase reporter assays

The wild type mouse STAT3 3′UTR luciferase reporter vectors were constructed by amplifying the mouse STAT3 mRNA 3′UTR and cloning it into the pMIR-REPORT™ Luciferase vector. Constructs with the ACTTACA to ACTAACT mutation at the pupative binding site was also generated and used as the control. RAW264.7 cells were cotransfected with 80 ng luciferase reporter plasmid, 40 ng thymidine kinase promoter-Renilla luciferase reporter plasmid, and the indicated miRNA mimics or controls (final concentration, 10 nM). After 24 h, luciferase activities were measured using the Dual-Luciferase Reporter Assay System (Promega), according to the manufacturer's instructions.

### RNA isolation and real-time quantitative PCR (qPCR)

Total RNA was extracted with TRIzol (Invitrogen). Real-time quantitative PCR, using SYBR Green detection chemistry, was performed on a 7500 Real-Time PCR System (Applied Biosystems) as we previously described [22]. For miRNA analysis, the reverse-transcriptase primer for miR-223 was 5′ -GTCGTATCCAGTGCAGGGTCCGAGGTATTCGCACTGGATACGACTGGGGT. QPCR primers were 5′-TGGCTGTCAGTTTGTCAAAT-3′ (forward) and 5′-GTGCAGGGTCCGAGGT-3′ (reverse). U6 small nuclear RNA was quantified using its reverse primers for reverse-transcriptase reaction and its forward and reverse primers for qPCR, which were 5′-CTCGCTTCGGCAGCACA-3′ (forward) and 5′-AACGCTTCACGAATTTGCGT-3′ (reverse). The relative expression level of miRNA was normalized by U6 expression. Annealing of miR-223 and U6 primers was done at 54°C. For IL-6, TNF-α and STAT3, annealing was done at 60°C. The data was normalized by the β-actin expression in each sample.

### RNA interference

The STAT3-specific siRNA were 5′-GGGUCUGGCUAGACAAUAUTT-3′ (sense) and 5′- AUAUUGUCUAGCCAGACCCTT-3′ (antisense). The scrambled control RNA sequences were 5′- UUCUCCGAACGUGUCACGUTT-3′ (sense) and 5′- ACGUGACACGUUCGGAGAATT-3′ (antisense). SiRNA duplexes were transfected into RAW264.7 cell, primary murine peritoneal macrophages or bone marrow derived macrophages at a final concentration of 30 nM.

### Immunoblotting

Total cell lysates were prepared and subjected to SDS/PAGE gel, transferred into PVDF membrane, and blotted as we previously described [23].

### Statistical analysis

All experiments were repeated three times. The data was presented as the mean ± SD. Statistical significance was determined by Student's *t* test, analysis of variance (ANOVA) using SPSS (v 10.0), with a p value less than 0.05 considered statistically significant.

## Results

### LPS and poly (I∶C) stimulation significantly induced the decrease in miR-223 expression in macrophages

**Figure 1:**
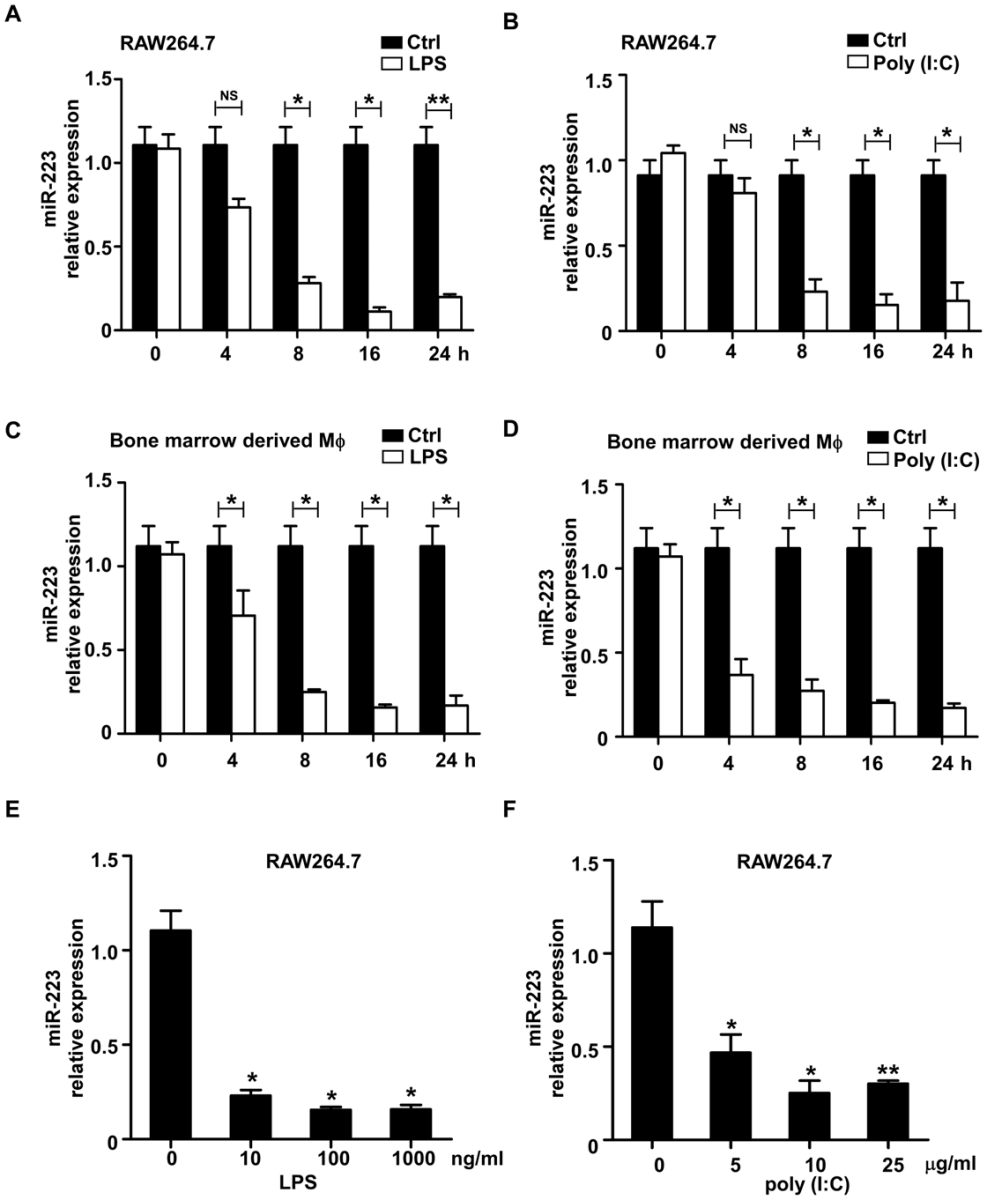
Down-regulation of miR-223 expression in RAW264.7 cells and primary macrophages stimulated with TLR agonists. (A–D) RAW264.7 and bone marrow-derived macrophages were stimulated with 100 ng/ml LPS or 10 µg/ml poly (I∶C) for the indicated time points. The expression of miR-223 was measured by q-PCR and normalized to the expression of U6. (E–F) RAW264.7 cells were stimulated with different concentrations of LPS (E) or poly (I∶C) (F) for 24 h. The expression of miR-223 was determined by qPCR and normalized to the expression of U6. Data are the mean ± SD (n = 3) of three independent experiments. *** p<0.001; ** p<0.01; * p<0.05.

Previous studies have demonstrated that LPS augments the expression of several microRNAs such as miR-155 and miR-146 [13]. To identify other microRNAs that are potentially involved in the regulation of TLR4- or TLR3-triggered inflammatory responses, we selected several miRNAs which are enriched in myeloid cells and analyzed their expression in LPS- or poly (I∶C)-stimulated RAW264.7 cells and primary macrophages via q-PCR. One of those miRNAs, miR-223, was slightly decreased at 4 h post-stimulation and was rapidly down-regulated during the next 4 h, reaching the minimum at 16 h to 24 h (Fig. 1A–B). In primary bone marrow-derived macrophages, a similar down-regulation pattern for miR-223 was observed (Fig. 1C–D). In addition, the expression of miR-223 was significantly decreased with lower concentrations of LPS or poly (I∶C) (Fig. 1E–F). The down-regulation of miR-223 upon stimulation of multiple TLR agonists suggests that miR-223 may function as a regulator of TLR-associated signaling events in macrophages.

### MiR-223 inhibits the production of IL-6 and IL-1β, but not TNF-α, in TLR-triggered macrophages

**Figure 2:**
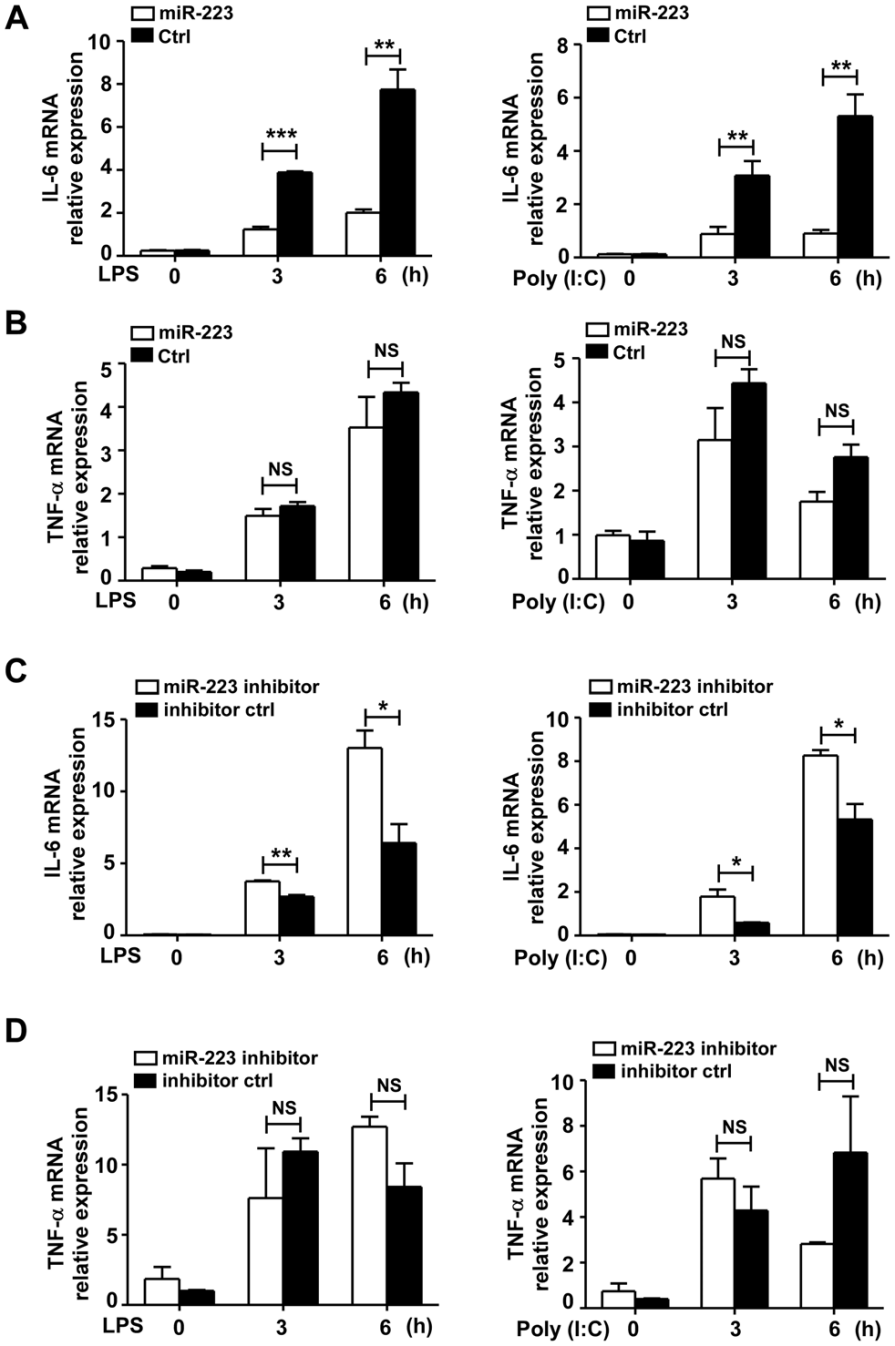
MiR-223 inhibits IL-6 mRNA expression but not TNF-α, in TLR-triggered macrophages. RAW264.7 cells were transfected with mimics or inhibitors of miR-223 and their controls at a final concentration of 30 nM. 24 h later, cells were stimulated with or without LPS (100 ng/ml) or poly (I∶C) (10 µg/ml). IL-6 (A, C) and TNF-α (B, D) mRNA levels were measured at 3 h and 6 h post-stimulation by qPCR. Data are the mean ± SD (n = 3) of three independent experiments. *** p<0.001; ** p<0.01; * p<0.05; NS, not significant.

**Figure 3:**
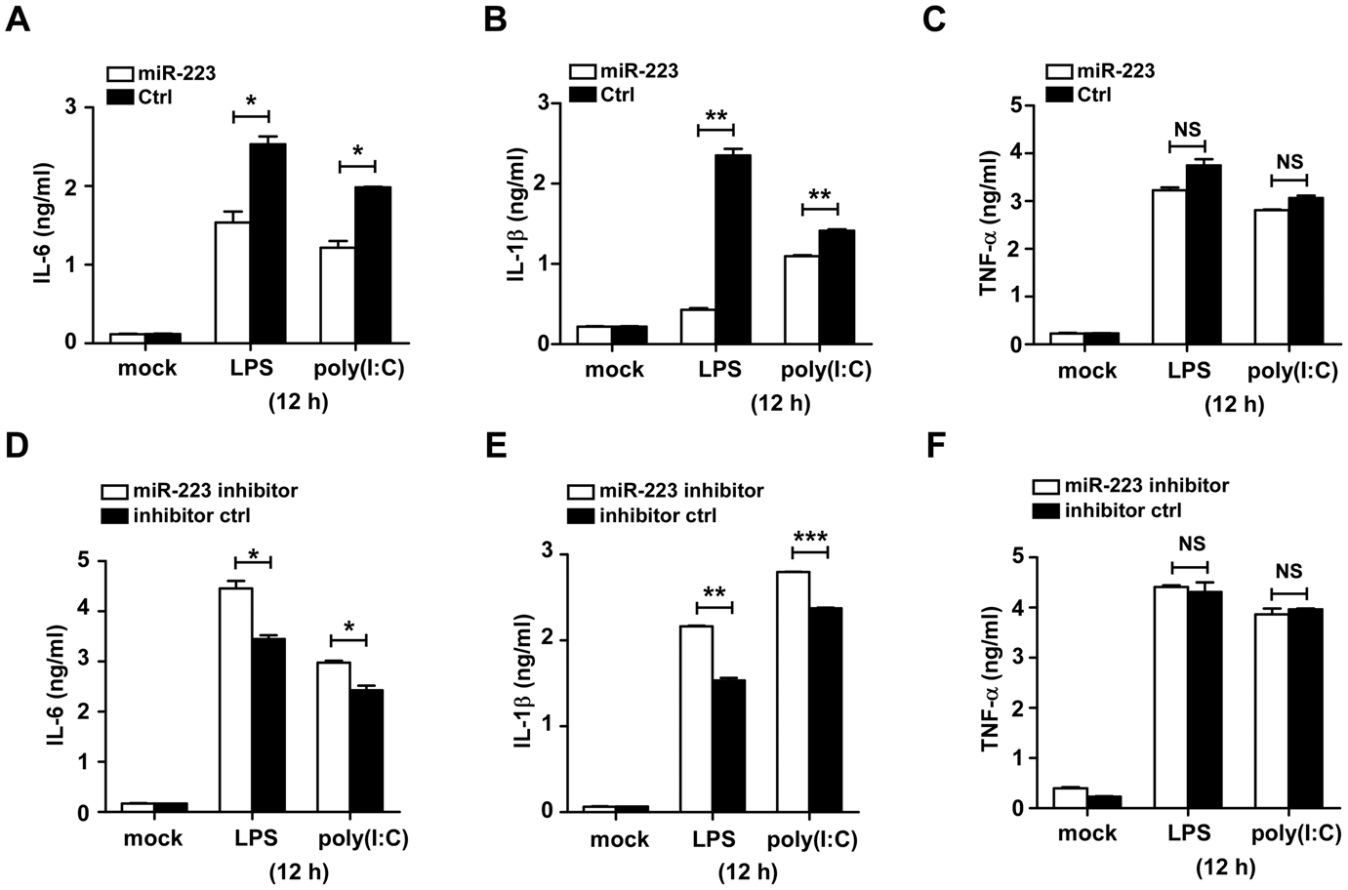
MiR-223 negatively regulates LPS or poly (I∶C)-triggered IL-6 and IL-1β production in macrophages. Raw264.7 cells were transfected with mimics or inhibitors of miR-223 and their controls at a final concentration of 30 nM. 24 h later, cells were stimulated with or without LPS (100 ng/ml) or poly (I∶C) (10 µg/ml). (A–F) Supernatants were collected after 12 h to measure IL-6, TNF-α, IL-1β by ELISA. Data are the mean ± SD (n = 3) of three independent experiments. *** p<0.001; ** p<0.01; * p<0.05; NS, not significant.

To further determine the functional significance of miR-223 down-regulation, RAW264.7 cells were transfected with miR-223 mimics to overexpress miR-223. The results showed that miR-223 mimics markedly decreased the mRNA and protein expression levels of IL-6 and IL-1β, but not TNF-α, production in RAW264.7 stimulated with LPS or poly (I∶C) (Fig. 2A, B and Fig. 3A–C). Conversely, increases in IL-6 and IL-1β expression were observed in RAW264.7 cells treated with anti-miR-223 (Fig. 2C, D and Fig. 3D–F), suggesting that LPS- or poly (I∶C)-induced decrease of miR-223 expression may be involved in the regulation of IL-6 and IL-1β production. Similarly, in primary peritoneal macrophages and bone marrow-derived macrophages stimulated with LPS, the miR-223 mimics markedly inhibited the production of IL-6 and IL-1β, but not TNF-α (Fig. S1). And the miR-223 antagomirs have a reverse effect on IL-6 and IL-1β production in peritoneal macrophages after LPS or poly (I∶C) stimulation (Fig. S2).

### MiR-223 negatively regulates the activation of STAT3 in macrophages

**Figure 4:**
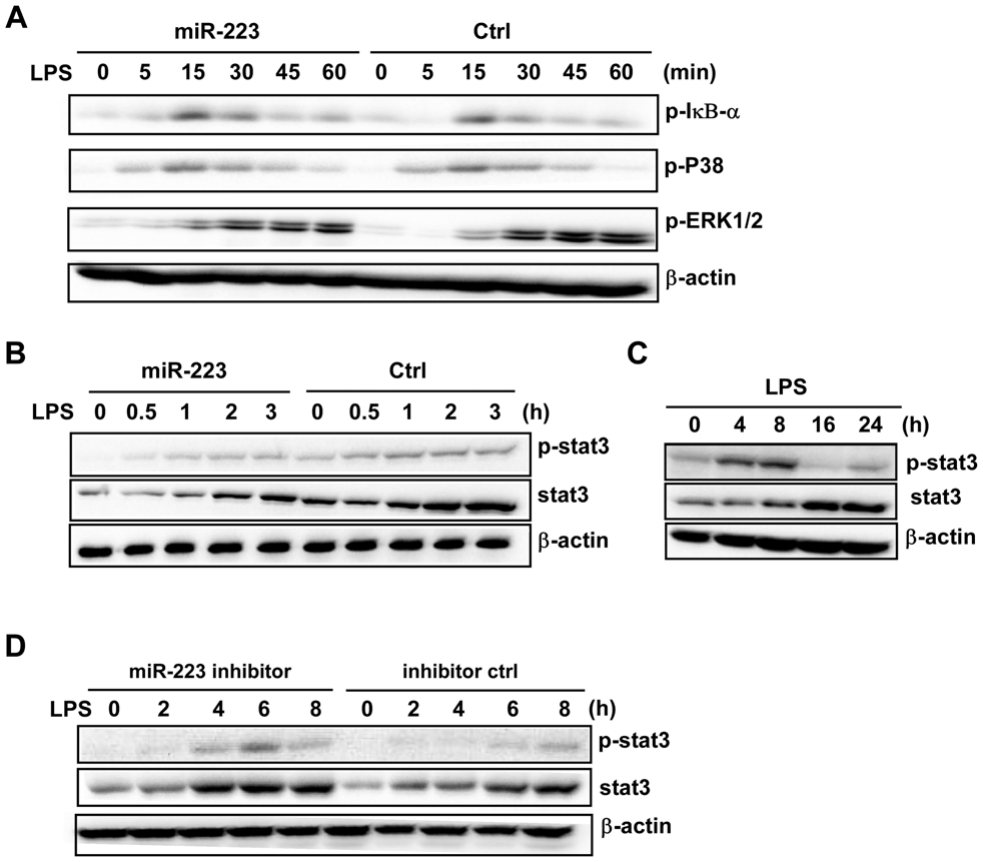
MiR-223 negatively regulates LPS-induced STAT3 activation in macrophages. (A) RAW264.7 cells were transfected with control and mimics of miR-223 at a final concentration of 30 nM, then stimulated with LPS (100 ng/ml) for the indicated time points. P-IκB-α, p-ERK1/2 and p-P38 were analyzed by immunoblotting. (B) RAW264.7 cells were treated as described above. Phosphorylated and total STAT3 were detected by immunoblotting. (C) RAW264.7 cells were stimulated with LPS for the indicated time points and STAT3 protein expression was measured by immunoblotting. (D) RAW264.7 cells were transfected with miR-223 inhibitors at a final concentration of 30 nM, and stimulated with LPS for the indicated time points, then the phosphorylated and total STAT3 protein were determined. The data shown represent three independent experiments.

After ligand binding, TLRs activate various intracellular signaling molecules involved in the NF-κB and MAPK pathway to promote cytokine production. We assessed the effect of miR-223 mimics on the expression levels of several components of the NF-κB and MAPK pathway in RAW264.7 cells. As shown in Figure 4A, over-expression of miR-223 did not influence the phosphorylation of IκBα, ERK1/2 or P38 upon LPS stimulation. Surprisingly, a decrease in the protein levels of phosphorylated and total STAT3 was seen in cells transfected with miR-223 mimics (Fig. 4B). LPS alone induced STAT3 tyrosine phosphorylation post-LPS challenge (Fig. 4C). However, the kinetic response of STAT3 phosphorylation with LPS challenge was altered in the presence of miR-223 inhibitors. RAW264.7 cells transfected with miR-223 inhibitors showed increased STAT3 protein and phosphorylation level as compared to the cells transfected with inhibitor control (Fig. 4D).

The STAT pathway has been recently reported to be important in regulating cytokine production towards TLR activation [7], [9]. To investigate the role of STAT3 in the LPS-triggered inflammatory response, we assessed the levels of total and phosphorylated STAT3 in RAW264.7 cells stimulated with LPS for longer time points. A significant increase in both total and phosphorylated STAT3 levels was detected (Fig. 4C). Since the LPS-induced decrease in miR-223 levels occurred at similar time points, our data suggests that miR-223 may function as a modulator of STAT3 protein.

**Figure 5:**
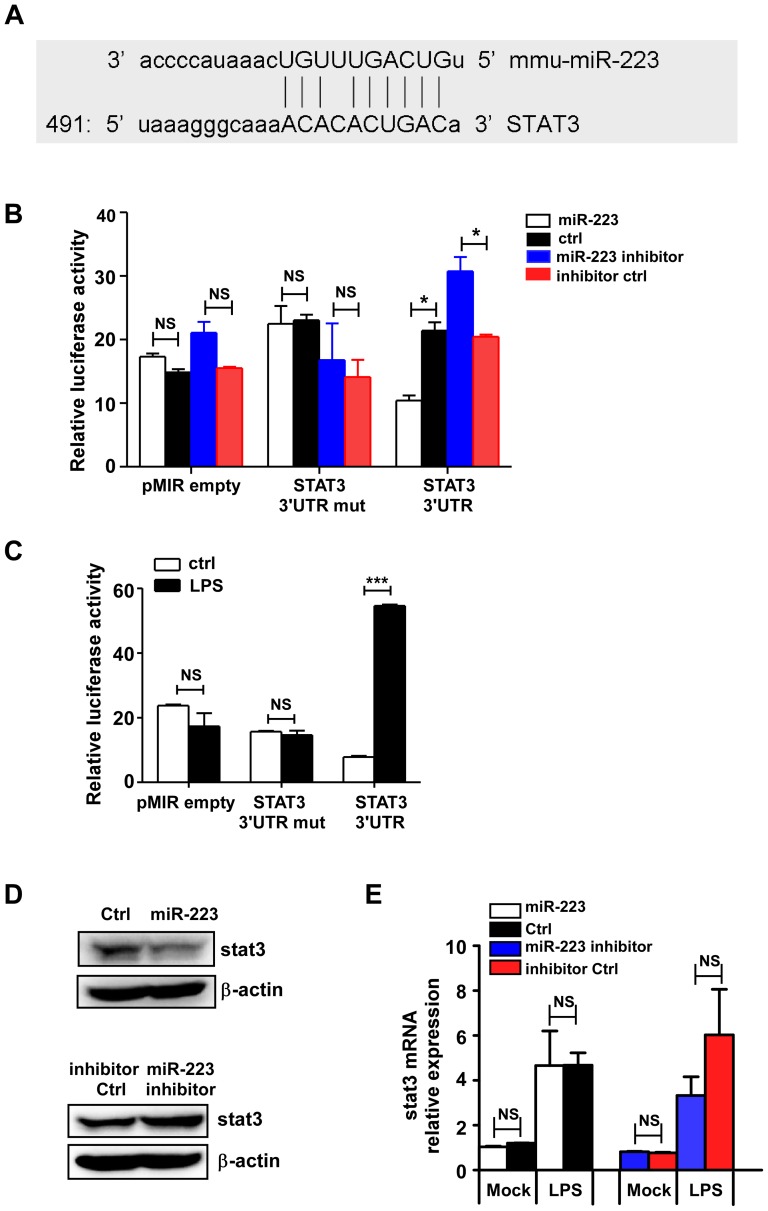
MiR-223 targets mouse STAT3 for translational inhibition but not mRNA degradation. (A) The alignment of miR223 and its target sites in 3′-UTR of STAT3 is shown. (B) RAW264.7 cells were cotransfected with pMIR-REPORT™-STAT3-3′UTR luciferase reporter plasmid, STAT3-3′-UTR-mutant or empty pMIR-REPORT™ and pTK-RL plasmid, together with miR-223 mimics or inhibitors (30 nM). After 24 h, firefly luciferase activity was measured and normalized by Renilla luciferase activity. (C) RAW264.7 cells were transfected with the pMIR-REPORT™ STAT 3′UTR, STAT 3′UTR mutant and control plasmids. Luceferase activity in cells with or without LPS stimulation was measured. (D) RAW264.7 cells were transfected mimics or inhibitors of miR-223 and their controls (30 nM). After 24 h, STAT3 protein expression was detected by immunoblotting. β-actin served as a loading control. The data shown represent three independent experiments. (E) RAW264.7 cells were transfected with mimics or inhibitors of miR-223 and their controls, 24 h later, STAT3 mRNA levels were determined with or without LPS at 12 h post stimulation. *** p<0.001; * p<0.05; NS, not significant. Data are the mean ± SD (n = 3) of one representative experiment. Similar results were obtained in three independent experiments.

Therefore, we hypothesized that STAT3 is a potential target of miR-223. Computational prediction via Targetsan 5.1 revealed that miR-223 was one of the non-conserved miRNAs that putatively target murine STAT3's 3′-UTR (Fig. 5A). Reporter plasmid was constructed by cloning the mouse STAT3 3′-UTR into the pMIR-REPORT™ luciferase vector, while the plasmid with the mutation at the pupative binding site was used as a control. RAW 264.7 cells were transfected with the plasmids in combination with miR-223 mimics or inhibitors. The results showed that miR-223 mimics markedly decreased, while miR-223 inhibitors significantly enhanced the luciferase activity in cells transfected with the STAT3 3′-UTR vector compared with the cells transfected with pMIR-empty plasmid. No change in luciferase activity was observed in cells transfected with the mutant STAT3 3′-UTR construct (Fig. 5B), indicating that STAT3 could be regulated post-transcriptionally by miR-223.

Furthermore, when RAW264.7 cells were firstly transfected with the STAT3-3′-UTR, STAT3-3′-UTR-mutant or pMIR-empty plasmids, the following LPS treatment for these cells significantly enhanced the luciferase activity of STAT3 3′-UTR plasmid but not the mutant or empty constructs (Fig. 5C). This result suggested that the endogenous down-regulated miR-223 might be responsible for the up-regulation of luciferase activity.

In addition, transfection with miR-223 mimics significantly down-regulated STAT3 protein expression in RAW264.7 cells, while an increase in STAT3 protein level was shown in RAW264.7 cells treated with miR-223 antagomirs (Fig. 5D). However, no significant change in STAT3 mRNA level was found between the control cells and the cells treated with miR-223 mimics or antagomirs at 12 h post-LPS stimulation (Fig. 5E), suggesting that STAT3 expression could be inhibited by miR-223 via translational inhibition but not mRNA degradation. Taken together, these results suggested that miR-223 can regulate STAT3 protein expression and that it is at least partially responsible for the increase in STAT3 protein expression observed in macrophages during stimulation by LPS.

### STAT3 siRNA decreases the production of IL-6 and IL-1β in macrophages stimulated with LPS and poly (I∶C)

Previous experiments have demonstrated that STAT3 plays an essential role in the signaling pathway for the production of these two important cytokines, IL-6 and IL-1β, which are co-regulated [24], [25].

**Figure 6:**
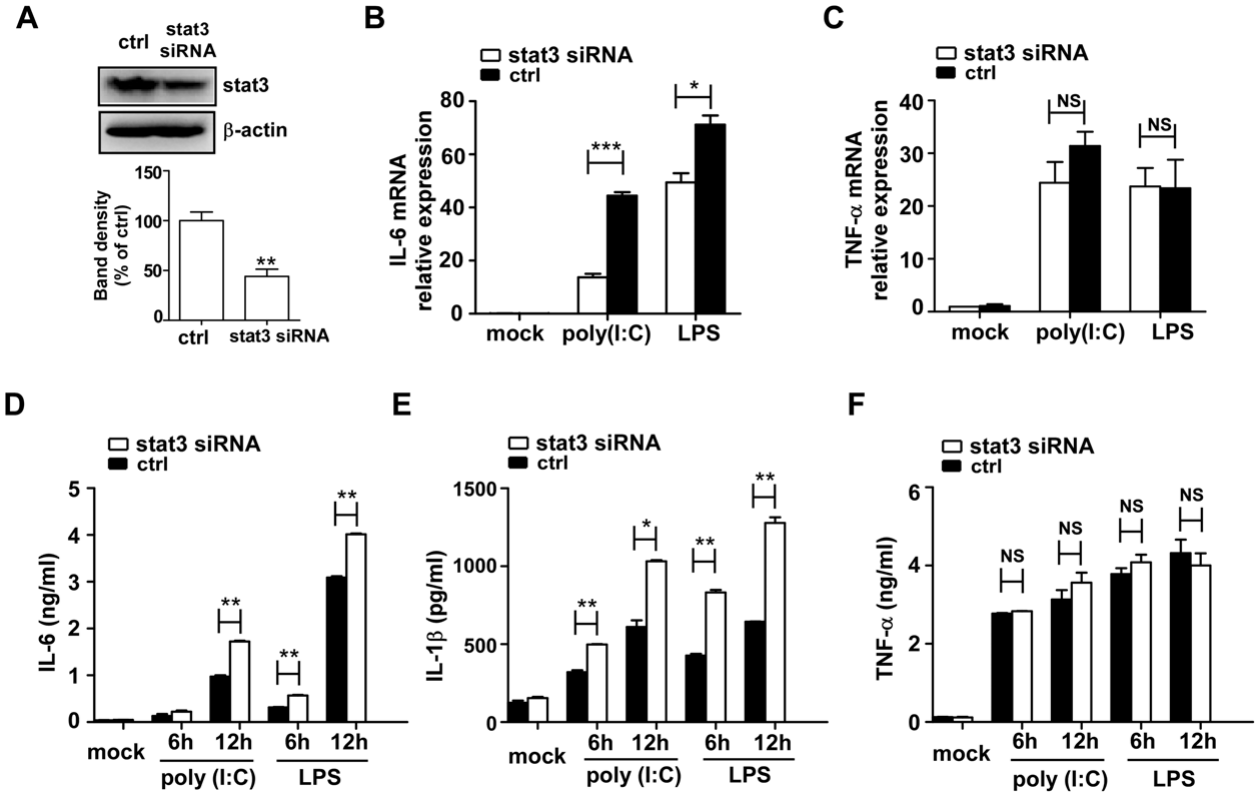
Knockdown of STAT3 inhibits LPS- and poly (I∶C)-induced IL-6 and IL-1β production. (A) RAW264.7 cells were transfected with STAT3 siRNA or control RNA at a final concentration of 30 nM. After 24 h, STAT3 protein expression was detected by immunoblotting and densitometry analysis was shown. The data shown represent three independent experiments. (B–C) STAT3 siRNA transfected RAW 264.7 cells were stimulated with poly (I∶C) (10 µg/ml) or LPS (100 ng/ml) for 6 h, mRNA levels of IL-6 and TNF-α were determined by qPCR. (D–F) The protein levels of the IL-6, TNF-α and IL-1β at the indicated time points were analyzed by ELISA. Data are the mean ± SD (n = 3) of one representative experiment. Similar results were obtained in three independent experiments. *** p<0.001; ** p<0.01; * p<0.05; NS, not significant.

To confirm the contribution of STAT3 on inflammatory cytokine generation, RAW264.7 cells were transfected with STAT3-specific small interfering RNA (siRNA). The siRNA down-regulated STAT3 protein level in RAW264.7 cells (Fig. 6A) and markedly inhibited LPS- or poly (I∶C)-induced mRNA and protein expression of IL-6 and IL-1β, but not TNF-α (Fig. 6B–F). The result was consistent with the effect of miR-223 over-expression on cytokine production. However, we were unable to detect differences in the levels of p-IκB-α, p-ERK1/2 and p-P38 between WT and STAT3-knockdown macrophages (data not shown).

### Transfection of miR-223 mimics to STAT3-siRNA pretreated cells abolishes its ability to inhibit IL-6 production

**Figure 7:**
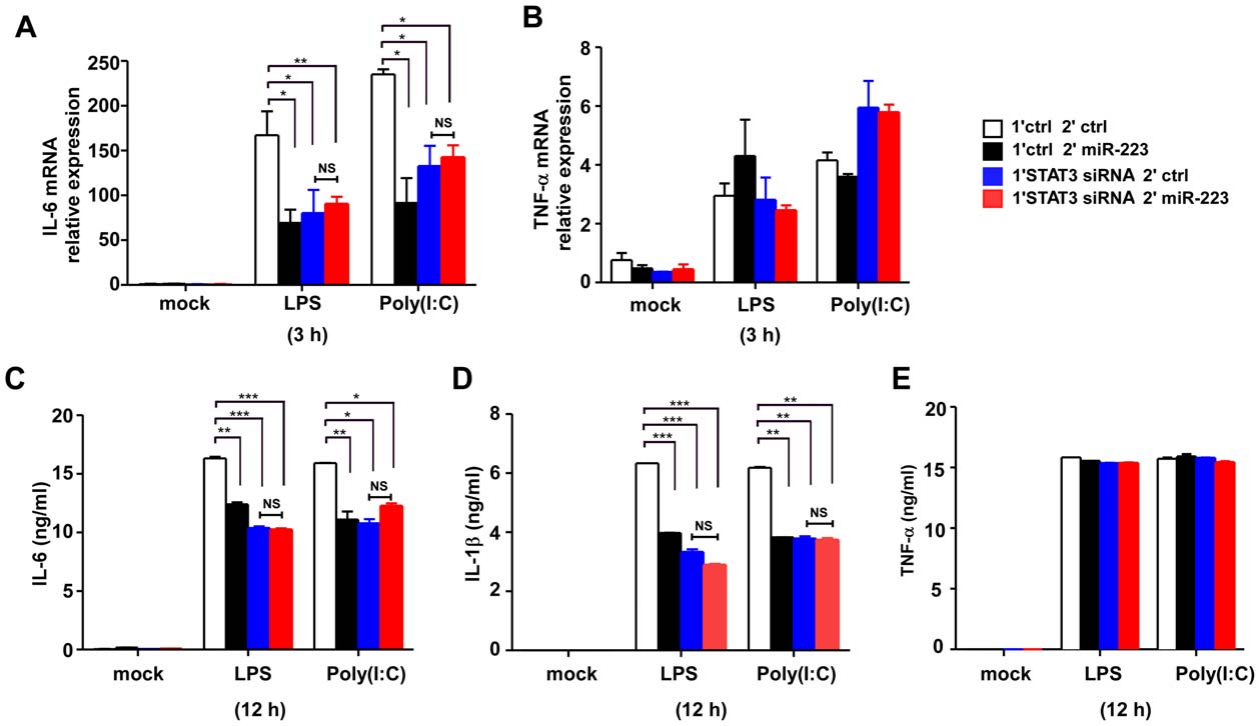
Transfection of miR-223 mimics to STAT3-siRNA pretreated cells abolishes its ability to inhibit IL-6 production. (A, B) RAW264.7 cells were first transfected with STAT3 siRNA or ctrl RNAs, 18 h later, cells were secondly transfected with ctrl or miR-223 mimics. After 24 h, cells were stimulated with LPS or poly (I∶C) for 3 h. IL-6 and TNF-α mRNA expression were analyzed by q-PCR. (C–E) Cells were treated as described in (A, B), IL-6, TNF-α and IL-1β production were measured by ELISA after 12 h stimulation with LPS or poly (I∶C). *** p<0.001; ** p<0.01; * p<0.05. Data are the mean ± SD (n = 3) of one representative experiment. Similar results were obtained in three independent experiments.

To further demonstrate a more direct causal relationship between miR-223 and STAT3, we first transfected RAW264.7 cells with STAT3 siRNA to knock down STAT3 expression, then miR-223 mimics or controls were transfected 18 h later. As shown in Fig. 7A, C and D, miR-223 mimics significantly decreased the production of IL-6 and IL-1β, but not TNF-α (Fig. 7B, E), in control siRNA pretreated RAW264.7 cells. However, miR-223 mimics has no effect on IL-6 or IL-1β secretion in STAT3-siRNA pretreated cells (Fig. 7A, C and D). Collectively, these results suggested that the effect of miR-223 to inhibit IL-6 and IL-1β production, but not TNF-α, was dependent upon targeting of STAT3.

### IL-6 down-regulates miR-223 after TLR ligand activation

TLR activation leads to the production of pro-inflammatory cytokines with quick activation of NF-κB and/or MAPK pathways. As mentioned above, we detected a significant decrease in miR-223 levels in RAW264.7 cells following LPS or poly (I∶C) stimulation for up to 24 h. To investigate the factors responsible for miR-223 down-regulation, several cytokines were tested. IL-6 was found to serve as a stimulatory factor in inducing the decrease in miR-223 levels.

**Figure 8:**
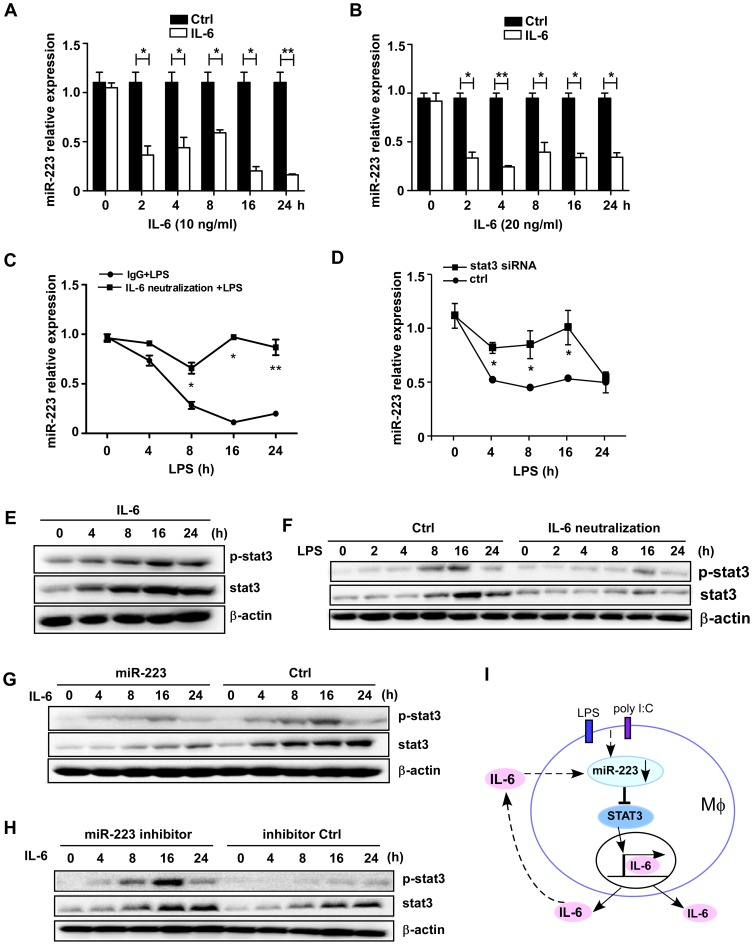
IL-6 acts as a main factor responsible for the down-regulation of miR-223 expression. (A–B) RAW264.7 cells were stimulated with 10 or 20 ng/ml of recombinant IL-6 for the indicated time points. The expression of miR-223 was determined by qPCR and normalized to the expression of U6. (C) Cells were treated with a mouse IL-6-specific blocking antibody or isotype control antibody (IgG1) upon LPS challenge. MiR-223 expression was determined by qPCR at the indicated time points. * p<0.05, ** p<0.01. (D) RAW264.7 cells were transfected with STAT3 siRNA at the final concentration of 30 nM. The expression of miR-223 was analyzed by q-PCR after LPS treatment for the indicated time. * p<0.05. (E) Cells were treated with mouse IL-6 (5 ng/ml) for the indicated time points and levels of total and phosphorylated STAT3 were analyzed by immunoblotting. (F) Cells were pretreated with IL-6 neutralization or IgG1 control antibody, STAT3 protein and phosphorylation levels were determined by immunoblotting at the indicated time points after LPS (100 ng/ml) stimulation. (G, H) RAW264.7 cells were firstly transfected with miR-223 mimics or inhibitors and their controls. After 24 h, cells were treated with IL-6 and levels of total and phosphorylated STAT3 were analyzed by immunoblotting. Data are representative of three independent experiments. (I) The proposed positive regulatory loop of IL-6/miR-223/STAT3 pathway in regulating the TLR-triggered inflammatory response.

Treatment of RAW264.7 with 10 or 20 ng/ml recombinant murine IL-6 significantly decreased the expression of miR-223, starting at the 0 to 2 h time-point, which is much earlier than that seen with LPS or poly (I∶C) challenge (Fig. 8A, B). Similarly, the stimulation of IL-6 (10 ng/ml) also induced the down-regulation of miR-223 expression in primary peritoneal macrophages (Fig. S3). To verify whether the TLR-triggered IL-6 is required for miR-223 down-regulation, RAW264.7 cells were pretreated with an IL-6-specific blocking antibody following LPS stimulation. As shown in Fig. 8C, no decrease in miR-223 was detected in the presence of an IL-6-specific blocking antibody compared with an immunoglobulin G1 (IgG1) isotype-matched control antibody upon LPS challenge, suggesting that LPS-induced miR-223 down-regulation is dependent on IL-6. We also transfected RAW264.7 cells with STAT3 siRNA and then exposed them to LPS for the indicated time followed by q-PCR analysis of miR-223 expression. It showed that STAT3 knockdown resulted in less decreased miR-223 expression (Fig. 8D). This is also an important piece of evidence indicating that secreted IL-6 could down-regulate miR-223 expression after LPS stimulation.

To elucidate the molecular mechanisms underlying the IL-6/miR-223/STAT3 axis-mediated regulation of TLR signaling, RAW264.7 were transfected with miR-223 mimics and treated with recombinant IL-6. IL-6 alone significantly induced STAT3 protein expression, up to the 24 h time point (Fig. 8E). As shown in Fig. 8F, pre-treatment of RAW264.7 with the IL-6-specific blocking antibody inhibited the activation of STAT3 by LPS, thus revealing that IL-6 signal transduction is mainly responsible for STAT3 induction downstream of TLR4. Additionally, we transfected RAW264.7 with miR-223 mimics and ctrl RNAs, and compared the STAT3 protein and phosphorylation levels after IL-6 challenge. The results showed that miR-223 significantly inhibited STAT3 augment and activation after IL-6 challenge (Fig. 8G). Conversely, an increase in STAT3 protein and phosphorylation level was observed in RAW264.7 cells treated with miR-223 antagomirs (Fig. 8H).

Collectively, these data demonstrated, for the first time, a causal role for miR-223 for regulating IL-6 signal transduction by directly targeting STAT3 in augmenting TLR-driven inflammatory responses. IL-6 induces miR-223 down-regulation and promoted the augment and activation of STAT3, which in turn facilitates the production of IL-6 and IL-1β, thus forming a positive regulatory loop for the production of pro-inflammatory cytokines (Fig. 8I).

## Discussion

In the present study, we reported that LPS or poly (I∶C) stimulation induced miR-223 down-regulation in a murine macrophage cell line and in primary macrophages. The change in miR-223 expression was accompanied by a substantial increase in STAT3 protein expression. Furthermore, forced expression of miR-223 was associated with a significant decrease in levels of STAT3 and a reduction in the production of IL-6 and IL-1β while miR-223 antagomirs exhibited a reverse effect in regulating IL-6 and IL-1β production. Similar effects were observed in cells transfected with STAT3 siRNA. These data suggest that miR-223 regulates STAT3 expression in TLR-triggered macrophages, a process that may be associated with the regulation of inflammatory responses in macrophages during microbial infection.

Another important finding of this study was the preferential up-regulation of IL-6 and IL-1β (compared with TNF-α) by miR-223/STAT3 pathway. Previous studies have also shown that blocking STAT3 activity preferentially inhibits LPS-mediated IL-1β and IL-6 production, but not TNF-α, in RAW264.7 cells [8], and STAT3 activation does not directly regulate LPS-induced TNF-α production in human monocytes [25]. The more current data demonstrated that genetically reducing the level of STAT3 activity in gp130^F/F^ mice reduced IL-6 expression in response to LPS and further supports a role for STAT3 in promoting IL-6 gene transcription [7]. Despite recent significant progress, the current understanding of the molecular basis underlying the regulation of the preference is still very limited. It is likely that the subtle differences in the transcriptional regulation of specific pro-inflammatory genes produced via TLR signaling cascades are accounted for the phenomenon. Our observations that STAT3 expression was up-regulated after LPS and IL-6 treatment are consistent with previous studies showing that long-term treatment with IL-6 increases STAT3 protein expression [26]. Another important question is how LPS or IL-6 functions to increase STAT3 protein expression. Most studies have focused on gene regulation at the transcriptional level, for example, some have shown that the STAT3 gene itself is activated by IL-6 signals, and an IL-6 response element (IL-6RE) exists within the STAT3 gene promoter, containing both a low affinity STAT3-binding element and a cAMP-responsive element (CRE), thus activates the STAT3 gene in cooperation with an unidentified CRE-binding protein [27]. In the present study, we found that IL-6 appears to not only activate transcription of STAT3 gene, but also to down-regulate miR-223 expression and, consequently, relieve the miR-223-mediated translational suppression of STAT3.

Macrophages initiate the innate immune response by recognizing pathogens, phagocytosing them, and secreting inflammatory mediators such as IL-6, IL-1β and TNF-α [28]. Increased IL-6 production is a hallmark of many human chronic inflammatory states, including sepsis, rheumatoid arthritis (RA), and inflammatory bowel disease (IBD)/colitis [29]–[31]. The diverse portfolio roles of IL-6 during the inflammatory response might be explained by its ability to initiate two modes of signaling: “classical” signaling via the interaction of IL-6 with its membrane-bound IL-6Rα subunit [32], and “trans-signaling” via a naturally occurring soluble IL-6Rα (sIL-6Rα) that is proteolytically cleaved from the cell surface [33]. In both scenarios, IL-6 exerts its action via the signal transducers gp (glycoprotein) 130, leading to the activation of the JAK/STAT and MAPK cascades [32]. In this study, we demonstrated that miR-223 may be involved in the IL-6 classical versus trans-signaling in regulating the TLR-driven inflammatory responses. The use of IL-6 instead of LPS or poly (I∶C) induced a quicker down-regulation of miR-223 expression. To further investigate the molecular mechanisms underlying the IL-6/miR-223/STAT3 axis-mediated regulation of TLR signaling, we pre-treated RAW264.7 cells with the IL-6 neutralization antibody and found it restored miR-223 expression and significantly diminished the LPS-induced increase in STAT3 expression. Pre-treatment with the IL-6-specific blocking antibody inhibited the activation of STAT3 by LPS, thus indicating that IL-6 signal transduction is mainly responsible for STAT3 induction downstream of TLR4. Additionally, miR-223 mimics significantly inhibited STAT3 augment and activation after IL-6 challenge, while an increase in STAT3 protein and phosphorylation level was observed in RAW264.7 cells treated with miR-223 antagomirs. Thus, it is likely that an increase in IL-6 production during LPS or poly (I∶C) stimulation serves two purposes: first, it directly activates STAT3 transcription through an IL-6 response element (IL-6RE) in the STAT3 gene promoter [27]; and second, it provides additional signals to down-regulate miR-223 expression, thus serving as a posttranscriptional gene regulation mechanism.

From the work presented here, we can now appreciate that the expression of IL-6 in response to LPS or poly (I∶C) initiates a positive feedback loop in which secreted IL-6 down-regulated miR-223 expression, leading secondarily to an increase in STAT3, which then drives the expression of IL-6 and IL-1β in a positive regulatory loop. However, the biological relevance of the ability of STAT3 to promote the production of IL-6 and IL-1β remains to be established. A study showed that the unphosphorylated STAT3 (U-STAT3) forms a complex with unphosphorylated NF-κB (U-NF-κB) and binds to the κB elements of promoters, such as the IL-6 gene, to induce their transcription [26]. In addition to the important role of U-STAT3, STAT3 tyrosine phosphorylation on residue 705 has recently been reported to be crucial for LPS-mediated IL-6 and IL-1β production [8]. Additionally, STAT3 is necessary for CXCR2-mediated neutrophil chemotaxis by regulating the amplitude of MIP-2-induced Raf, mitogen-activated protein kinase/extracellular signal-regulated kinase (MEK), and extracellular signal-regulated kinase (ERK) signaling [34], which suggests that STAT3 can positively regulate ERK signaling in neutrophils. STAT3 is constitutively activated in nearly all human cancers and it is associated with various transcriptional activities in the nuclei [5]. The study of STAT3 in cancer has revealed that JAK2/STAT3 was activated in starved Hela cells, with the activated STAT3 directly bound to IL-6 promoter to increase IL-6 mRNA and protein secretion [35]. All of the previous work confirmed that the concentration of total STAT3 protein is essential for the activation of IL-6 or IL-1β expression, though the mechanisms may be distinct from that used by P-STAT3. In our present work, we demonstrated that the status of tyrosine phosphorylation on residue 705 of STAT3 is consistent with total STAT3 protein expression, both of which may be required for IL-6 and IL-1β production.

Taken together, our findings provide a new explanation characterizing the molecular mechanism responsible for the regulation of IL-6 production after TLR-triggered macrophage activation. It may provide useful insights to the understanding of molecular mechanisms by which the IL-6/miR-223/STAT3 pathway controls the inflammatory response and promotes the pathogenesis of inflammatory diseases.

## Supporting Information

Figure S1 The effect of miR-223 on the production of IL-6 and IL-1β in primary macrophages. Mouse peritoneal macrophages (A, B, C) or bone marrow derived macrophages (D, E, F) were transfected with miR-223 mimics or control at a final concentration of 30 nM. 24 h later, cells were stimulated with 100 ng/ml of LPS. The production of IL-6, TNF-α and IL-1β were analyzed by ELISA. The data shown represent three independent experiments. ** p<0.01; * p<0.05; NS, not significant
(TIF)

Figure S2 The effect of miR-223 antagomirs on IL-6 and IL-1β production in peritoneal macrophages. Mouse peritoneal macrophages were transfected with miR-223 inhibitors or control at a final concentration of 30 nM. 24 h later, cells were stimulated with LPS (100 ng/ml) or poly (I∶C) (10 µg/ml). The mRNA (A–D) and protein (E–F) expression of IL-6, TNF-α and IL-1β were analyzed by q-PCR and ELISA assay. ** p<0.01; * p<0.05; NS, not significant, the data shown represent three independent experiments.
(TIF)

Figure S3 The down-regulation of miR-223 expression in peritoneal macrophages upon the stimulation of IL-6. Mouse peritoneal macrophages were treated with IL-6 at a final concentration of 10 ng/ml for the indicated time points, the expression of miR-223 was determined by qPCR and normalized to the expression of U6 in each sample. Data were representative of three independent experiments. * p<0.05
(TIF)
