# Supplementary figures and images for: Inducible MicroRNA-223 Down-Regulation Promotes TLR-Triggered IL-6 and IL-1β Production in Macrophages by Targeting STAT3
Source: PLoS One. 2012 Aug 24;7(8):e42971. doi: 10.1371/journal.pone.0042971 (PMC3427313; doi:10.1371/journal.pone.0042971)

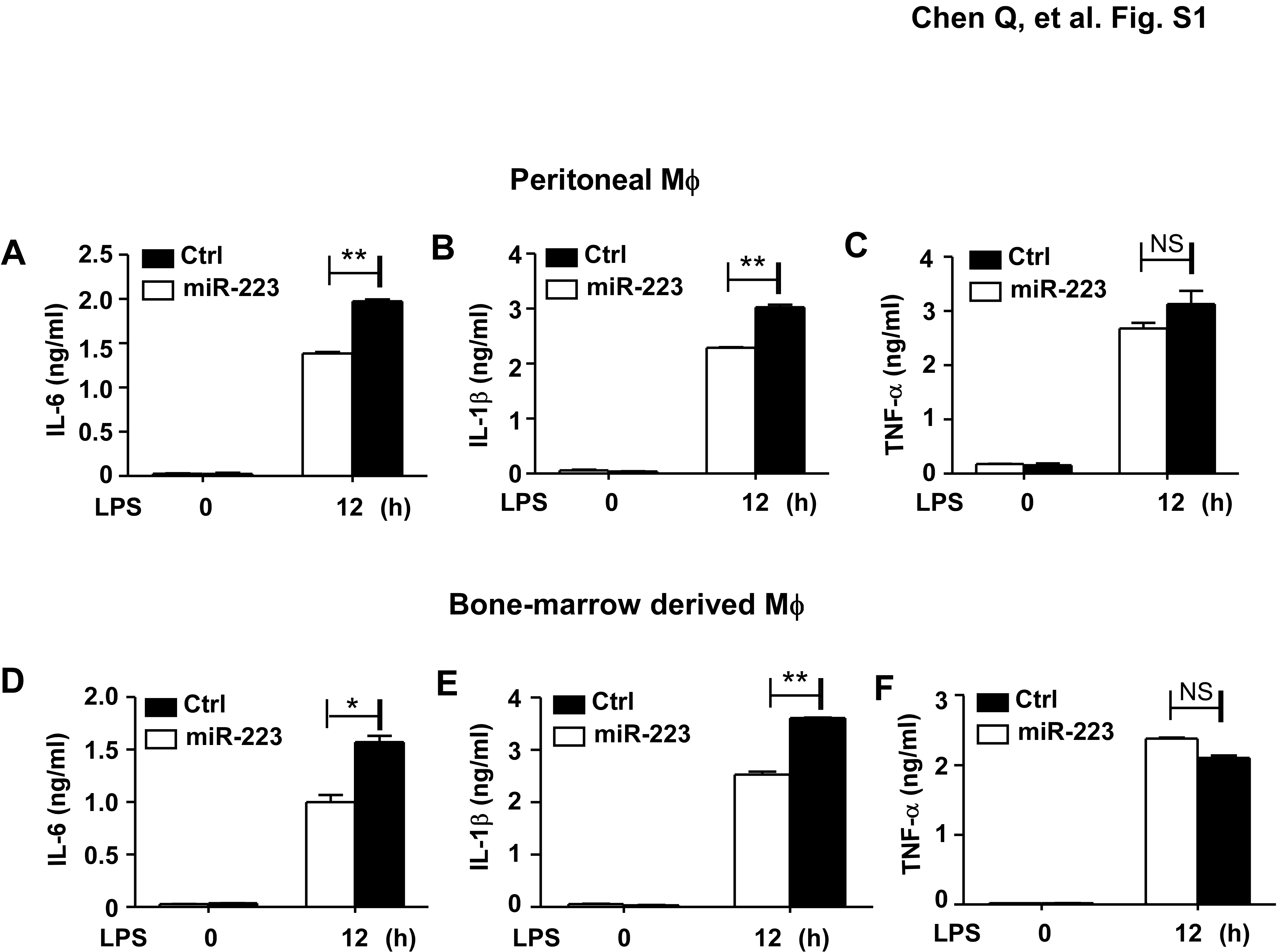

Supplement: Figure S1 — The effect of miR-223 on the production of IL-6 and IL-1β in primary macrophages. Mouse peritoneal macrophages (A, B, C) or bone marrow derived macrophages (D, E, F) were transfected with miR-223 mimics or control at a final concentration of 30 nM. 24 h later, cells were stimulated with 100 ng/ml of LPS. The production of IL-6, TNF-α and IL-1β were analyzed by ELISA. The data shown represent three independent experiments. ** p<0.01; * p<0.05; NS, not significant (TIF) [file pone.0042971.s001.tif]

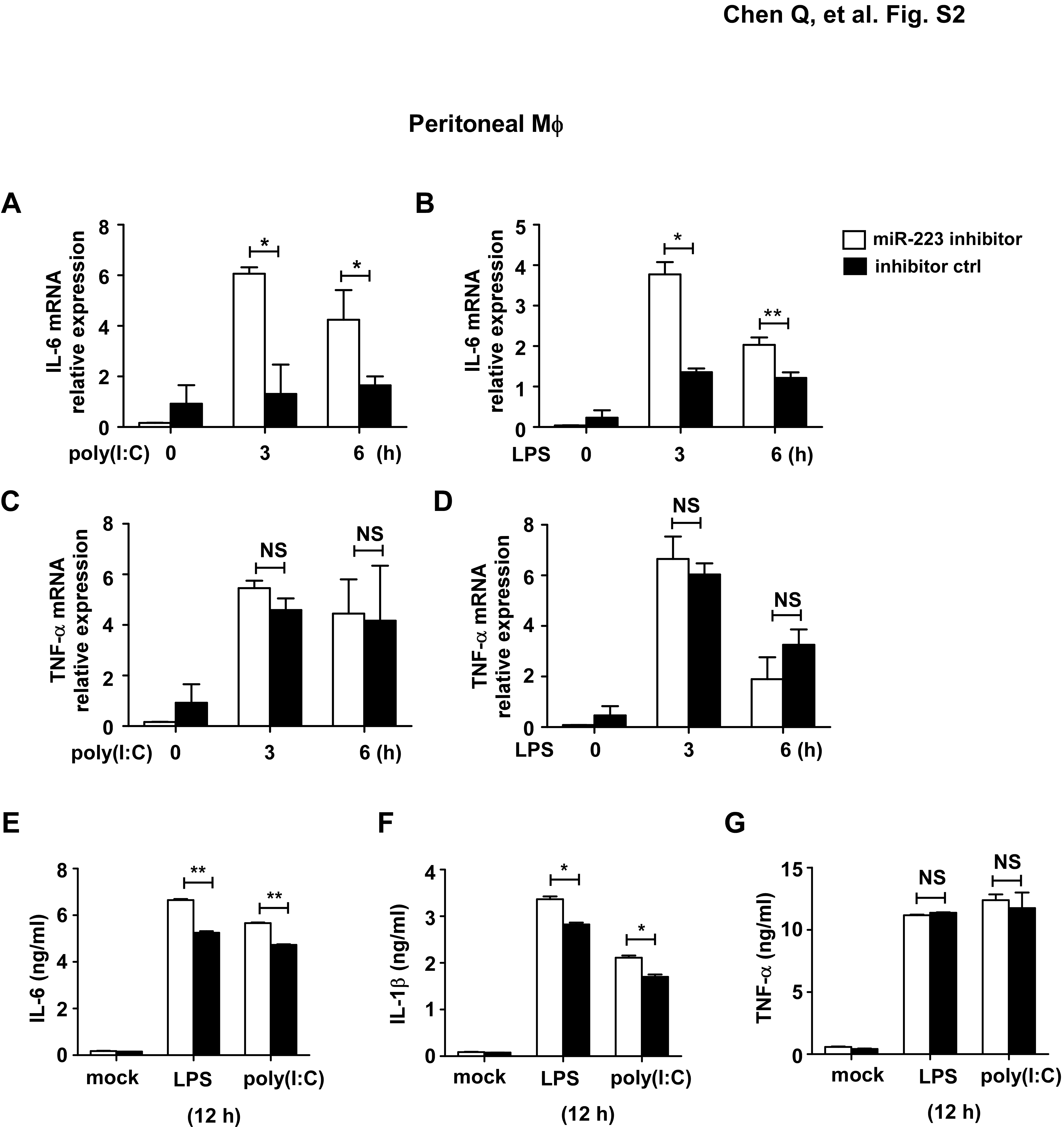

Supplement: Figure S2 — The effect of miR-223 antagomirs on IL-6 and IL-1β production in peritoneal macrophages. Mouse peritoneal macrophages were transfected with miR-223 inhibitors or control at a final concentration of 30 nM. 24 h later, cells were stimulated with LPS (100 ng/ml) or poly (I∶C) (10 µg/ml). The mRNA (A–D) and protein (E–F) expression of IL-6, TNF-α and IL-1β were analyzed by q-PCR and ELISA assay. ** p<0.01; * p<0.05; NS, not significant, the data shown represent three independent experiments. (TIF) [file pone.0042971.s002.tif]

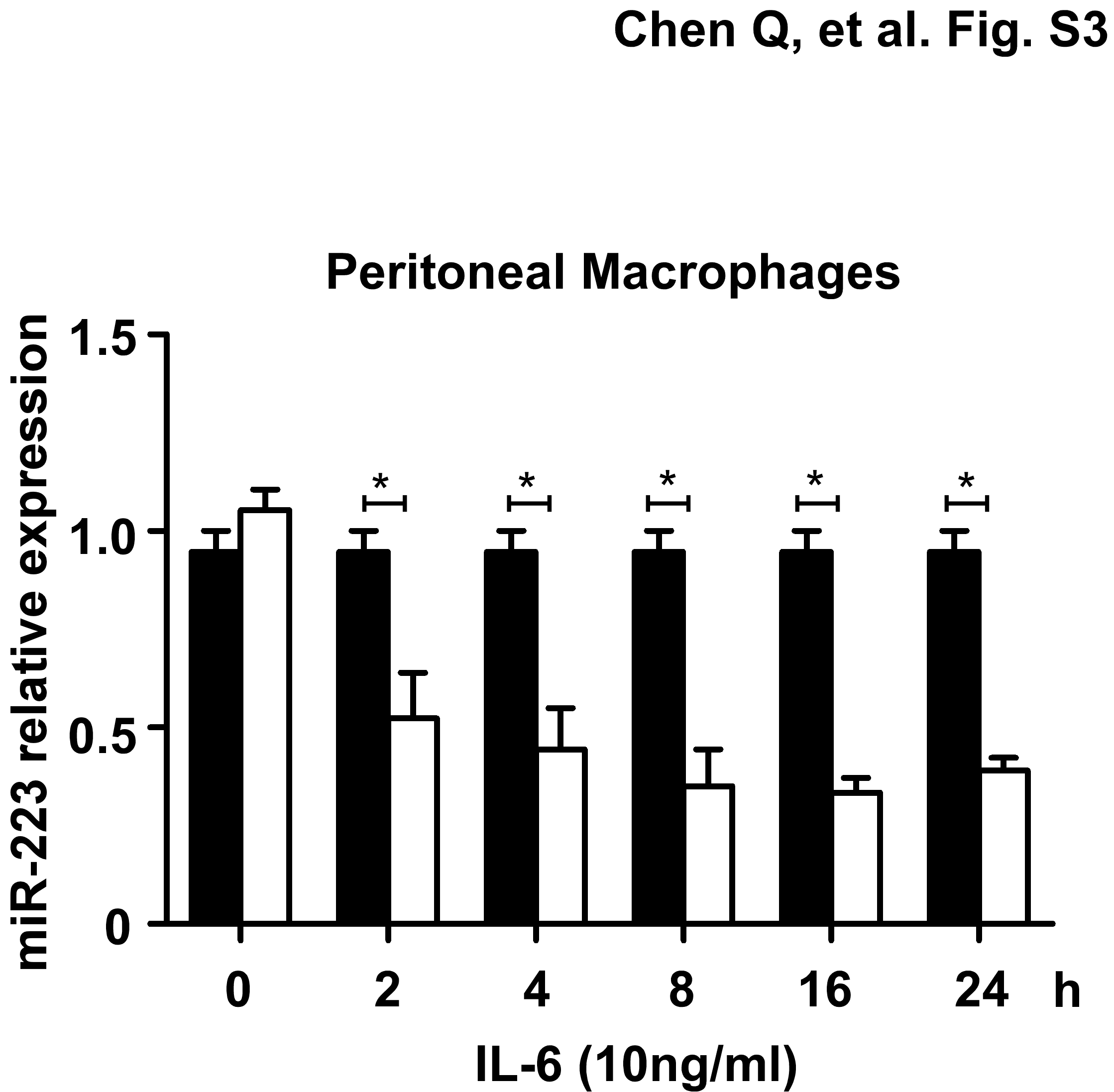

Supplement: Figure S3 — The down-regulation of miR-223 expression in peritoneal macrophages upon the stimulation of IL-6. Mouse peritoneal macrophages were treated with IL-6 at a final concentration of 10 ng/ml for the indicated time points, the expression of miR-223 was determined by qPCR and normalized to the expression of U6 in each sample. Data were representative of three independent experiments. * p<0.05 (TIF) [file pone.0042971.s003.tif]
